# Assessment of Child Immunization Coverage and Associated Factors with Full Vaccination among Children Aged 12–23 Months at Mizan Aman Town, Bench Maji Zone, Southwest Ethiopia

**DOI:** 10.1155/2017/7976587

**Published:** 2017-12-24

**Authors:** Asrat Meleko, Mesfin Geremew, Frehiwot Birhanu

**Affiliations:** Department of Public Health, College of Medicine and Health Sciences, Mizan-Tepi University, Mizan Teferi, Ethiopia

## Abstract

Immunization remains one of the most important and cost-effective public health interventions to reduce child mortality and morbidity. Globally, it is estimated to avert between 2 and 3 million deaths each year. In Ethiopia, immunization coverage rates stagnated and remained very low for many years. Thus, this study was aimed to assess child immunization coverage and factors associated with full vaccination among children aged 12–23 months in Mizan Aman town. The study design was community-based cross-sectional survey. Data was collected by using pretested structured questionnaire. A total of 322 mothers/caretakers were interviewed. Based on vaccination card and mothers/caretakers' recall, 295 (91.6%) of the children took at least a single dose of vaccine. From total children, 27 (8.4%) were not immunized at all, 159 (49.4%) were partially immunized, and 136 (42.2%) were fully immunized. Mothers/caretakers educational level, fathers' educational level, place of delivery, maternal health care utilization, and mothers/caretakers knowledge about vaccine and vaccine-preventable disease showed significant association with full child immunization. The finding from this study revealed that child immunization coverage in the studied area was low. Thus the town health office and concerned stakeholders need to work more to improve performance of the expanded program on immunization in this area.

## 1. Introduction

Universal immunization of children against six preventable diseases (tuberculosis, diphtheria, Pertussis, tetanus, polio, and measles) is crucial to diminish childhood mortality and morbidity across the world. Unquestionably improving access to and utilization of routine immunization services are the best option for the prevention and control of vaccine-preventable diseases (VPD). As a consequence, the expanded program on immunization (EPI) was launched in 1974 as a global program for controlling and reducing death from vaccine-preventable diseases [1].

Immunization is considered as one of the most powerful and cost-effective of all health interventions. It also believed to prevent debilitating illness and disability and saves millions of lives every year [2]. For instance, immunization currently forestalls an estimated two to three million deaths every year in all age groups from diphtheria, tetanus, pertussis (whooping cough), and measles. Moreover immunization contributes a lot for a given nation by reducing the risk of disability from infectious diseases such as poliomyelitis [3]

Even though vaccines were widely regarded as an effective tool to halt the burden associated with vaccine-preventable disease (VPD), across the world 26.3 million children below the age of one year had not been immunized with diphtheria-tetanus-pertussis vaccine (DTP3) in 2008 [4]. A recent report from world health organization (WHO) revealed that the number of children under one year of age who did not receive diphtheria-tetanus-pertussis vaccine (DTP3) vaccine worldwide was estimated to be 21.8 million in 2013 compared to 22.8 million in 2012. Almost seventy percent of these children live in ten countries and more than half of them are found in Africa including Ethiopia, Kenya, and South Africa. In 2016, routine immunization services such as DTP3 vaccine did not reach an estimated 19.5 million infants worldwide. Despite this recent success, more than 3 million people die from vaccine-preventable diseases each year. Approximately 1.5 million of these deaths are in children less than 5 years old from diseases that can be prevented by immunization [5, 6].

Various reports indicated that the death of children is more common in developing world. Particularly children living below sub-Saharan African die every year due to communicable diseases that can be prevented by immunization. For instance, out of 9 million deaths of children globally as a result of vaccine-preventable disease, a bigger proportion occurred in sub-Saharan Africa which was 4.4 million [4]. This is substantially attributed to inadequate immunization coverage and challenges in sub-Saharan Africa. Because in many parts of Africa vaccine infrastructure has been suboptimal, especially for routine vaccination which is identified as the main factor for under vaccination [7]. A report from WHO revealed that around 60% of children's who were not reached with routine immunization services are from 10 countries where majority are from sub-Saharan African countries [5]. And five of those African regions including Ethiopia were the region that continues to even increase further the pool of unimmunized children [8].

Even though, in 1980, the government of Ethiopia initiated the implementation of EPI with goal of increasing vaccination coverage against the six childhood killer diseases by 10% each year to reach 100% coverage in 1990, this goal has largely remained unrealized even using different efforts [9]. As a result an estimated 472,000 children still die each year before their fifth birthday largely from vaccine-preventable diseases. Despite the high prevalence of vaccine-preventable diseases (VPDs) in the country, immunization coverage rates stagnated and remained very low for many years. The Ethiopia Demographic Health Survey (EDHS) 2011 showed coverage level for third dose of diphtheria-tetanus-pertussis vaccine (DTP3) and that of full immunization at country level were to be 36.5% and 24.3%, respectively [10]. Also the EDHS 2016 revealed that close to two in every five children aged 12–23 months (39%) received all basic vaccinations at some time, and 22% were vaccinated by the appropriate age. It is found that the percentage of children aged 12–23 months who are fully vaccinated increased by 15%, from 24% in 2011 to 39% in 2016 [11]. Even if there are certain discrepancies on immunization coverage across regions, the findings in Southern Nations, Nationalities and Peoples' Region (SNNPR) have similar percentage with other parts of the country.

It is known that the World Health Assembly in May 2012 endorsed the Global Vaccine Action Plan (GVAP) as a roadmap to prevent millions of deaths through more equitable access to vaccines. Under this plan countries hope to achieve vaccination coverage of at least 90% nationally and at least 80% in each district by 2020 [3]. Thus the government of Ethiopia is expected to do more to achieve GVAP plan. It is fact that, to amplify the child immunization coverage in the study area and as a whole within the country, the root causes why they do not immunize their children should be known. But in Mizan Aman town there was shortage of data which reveals child immunization coverage and factors associated with complete vaccination. Thus, this study would fill the existing information gap and help policy makers, program planning bodies, and service providers to get rid of the obstacles and improve child immunization to attain intended control of vaccine-preventable diseases. Also it would be a guide for effective utilization of resources by providing relevant and evidence-based recommendations for addressing issues related to vaccination.

## 2. Objectives

### 2.1. General Objective

To assess the immunization coverage of children aged 12–23 months and associated factors with full vaccination in Mizan Aman town, Bench Maji Zone, Southwest Ethiopia.

### 2.2. Specific Objectives

- To determine child immunization coverage for the eight doses of vaccine among children aged 12–23 months in Mizan Aman town, Bench Maji Zone, Southwest Ethiopia
- To identify factors associated with full vaccination of children in Mizan Aman town, Bench Maji Zone, Southwest Ethiopia

## 3. Method and Materials

### 3.1. Study Area and Design

A community-based cross-sectional study design was conducted from April 11 to May 1, 2017. The study was conducted in Mizan Aman town, one of the 11 Woreda found in Bench Maji zone, South West Ethiopia. Mizan is the capital city of Bench Maji zone, located at 560 km to south of Addis Ababa. There are 5 kebeles (small administrative units) in Mizan Aman town. The total population in Mizan Aman town is 50,113 of this 24555 are male and 25558 are female (in 2011). The estimated number of eligible children for this study was 1452. There are one general hospital, one health center, two health posts, 12 junior private clinics, 7 medium private clinics, and 20 drug stores in Mizan Aman town.

### 3.2. Source Population

All children aged 12 to 23 months with their mothers/caretakers living in Mizan Aman town were the source population.

### 3.3. Study Population

Children aged 12 to 23 months with their mothers or caretakers that will be sampled from source population were the study population.

### 3.4. Sample Size Determination

The sample size required was determined based on single proportion population formula with the assumption of 5% margin of error (*d*), 95% confidence level (*Z*), and the immunization coverage assumed to be 39% [11] by history and vaccination card which is taken from EDHS 2016, to get the possible sample size. The sample size was calculated as follows:

(1) n=Zα/22P1−Pd2,n=1.9620.391−0.390.052,n=365,

where

 
*P* is proportion of immunization coverage (39%);
 
*d* is margin of error = 0.05;
 
*Z*_*α*/2_ is confidence level required and *Z*_*α*/2_ at 95% CI = 1.96;
 
*n* is minimum sample size.

Since the total eligible children for this study in study area was less than 10,000 which was 1452, by using population correction formula the sample size became 293:

(2) nf=ni1+ni/nt,nf=2931+293/1452=293,

where

 
*n*_*i*_ is the initial sample size (calculated sample size);
 
*n*_*f*_ is the final sample size;
 
*N*_*t*_ is the total no of eligible children in two kebeles.

Then by considering 10% nonresponse rate the final sample size was 322.

### 3.5. Sampling Technique

Multistage cluster sampling was used to take the appropriate sample. Initially, Mizan Aman town was classified into two clusters based on residents. Then, one kebele (small administrative unit in the country) from each cluster was selected with a total of two kebeles from all clusters using lottery method. The total sample size was allocated proportionally to each kebele depending on the total number of children aged 12 to 23 months.

The sampling frame was obtained from health extension workers registration books. In both kebeles, the first household was selected randomly from the central location of the kebele. The subsequent household was selected according to the inclusion criteria based on the principle of next nearest household. Households in the kebele were visited until the proportionally allocated sample size for both kebeles was fulfilled. One child was selected randomly from those households having two and more children.

### 3.6. Inclusion Criteria and Exclusion Criteria

#### 3.6.1. Inclusion Criteria

- Children with their mothers or caretakers living in Mizan Aman town for at least 12 months before the date of data collection and child aged between 12 and 23 months were included in this study.
- Caretakers aged 15 years and above were included in this study.
- Mothers/caretakers able to be interviewed were included in this study.

#### 3.6.2. Exclusion Criteria

- Children with their mothers or caretakers who had not been living in the study area for at least 12 months on the date of data collection were excluded from the study.
- Caretakers aged below 15 years were excluded from the study.

### 3.7. Data Collection Procedure

An interviewer-administered structured questionnaire was used to obtain the required data. The instrument was constructed from a review of available literature on immunization coverage, WHO questionnaire [12], and EDHS for immunization coverage [10] and was translated into local language. Data was collected by 4th year graduating public health students.

Households (mothers/caretakers) with eligible children in both kebeles were visited by data collectors as stated in sampling technique until the proportionally allocated sample size in each kebele was achieved. Mothers or caretakers were asked to show immunization cards, and then vaccines received were copied. For those mothers/caretakers who had no vaccination card, different appropriate questions were asked in order to determine the vaccination status of the child for each specific vaccine. In case of pentavalent and polio vaccine, the mothers were asked to report the number of pentavalent/polio vaccines that the child had received. In order to reduce recall bias for mothers/caretakers history, remainder such as site of administration (whether it is taken as injection or orally, presence of scar, and also at what age they vaccinate) was included in instruments.

### 3.8. Data Quality Control

Questionnaire was prepared initially in English by the investigators and then translated to Amharic and another translator retranslates to English to check its consistency. Before the actual data collection, the questionnaire was pretested on 5% of children whose age was between 12 and 23 months in Shey Bench woreda, seiz town. After pretesting, necessary amendments were made accordingly, including on ambiguities of the questions, wording, logic sequence, and skipping order. Data collectors were trained on the study instrument and data collection procedure before data collection time. The collected data was checked for completeness and corrective measures were taken accordingly. The collected data was cleaned, coded, and explored before analysis.

### 3.9. Operational Definitions

#### 3.9.1. Fully Vaccinated/Complete Immunization

A child aged 12–23 months who received one dose of (Bacillus Calmette-Guérin) BCG, one dose of measles, three doses of DPT-HepB-Hib (pentavalent), and OPV by card plus mothers history was considered to be fully vaccinated.

#### 3.9.2. Partially/Incompletely Immunized

A child aged 12–23 months who had missed at least one dose of the eight vaccines was considered to be partially/incompletely immunized.

#### 3.9.3. Not Immunized

A child aged 12–23 months who did not received any vaccine before this study was considered to be not immunized.

#### 3.9.4. Coverage by Card Only

Coverage by card only meant coverage calculated with numerator based only on documented dose, excluding the numerator of those vaccinated by history.

#### 3.9.5. Coverage by Card plus History

Coverage by card plus history meant coverage calculated with numerator based on card and mother's report.

#### 3.9.6. Good Knowledge

Six knowledge questions were asked and correct answers were given score 1 and incorrect answers score 0. Those having scored greater than the mean were classified as having good knowledge.

#### 3.9.7. Poor Knowledge

Six knowledge questions were asked and correct answers were given score one and incorrect answers score 0. Those having scored less than mean were classified as having poor knowledge.

#### 3.9.8. Caretakers

Caretaker is the most responsible person that provides care for the child that has no mother due to different reasons (death, separated from husband, and others).

#### 3.9.9. Index Child

Index child refers to a child that is included in the study from a household to have information on the demographic and immunization status.

#### 3.9.10. Literate

Mothers/caretakers/fathers with formal education or able to read and write are considered literate.

### 3.10. Data Analysis

Data was entered to SPSS version 21 after checking for completeness, then cleaned, and analyzed accordingly. Descriptive and analytical statistics including bivariate and multivariable analysis were done. Bivariate and multivariate analyses were used to examine association between dependent (full vaccination) and independent variables (sociodemographic variables, characteristics of child, etc.); a corresponding *p* value of <0.05 was considered to be statistically significant. To identify the independent factor that influences immunization completion, multivariable logistic analysis was carried out.

### 3.11. Study Variables

#### 3.11.1. Dependent Variable

- Full vaccination/complete immunization

#### 3.11.2. Independent Variables

- Sociodemographic characteristic of mother/caretaker (age, marital status, religion, occupation, educational status, and family income)
- Knowledge of mother on vaccine and vaccine-preventable disease
- Maternal health care utilization
- Time of travel to reach nearest health facility
- Characteristics of the child (age, birth order, and place of delivery)

### 3.12. Ethical Consideration

Permission to undertake the study was obtained from every relevant authority in Mizan Aman town and respective kebeles. Pertinent consent form and the information sheet were duly integrated along with the respective data collection instruments. All the study participants were clearly informed about the objective, benefits, and significance of the study as it has no harm. Finally, verbal informed consent was obtained from each study participant before the interview.

## 4. Results

### 4.1. Sociodemographic Characteristics of the Study Population

A total of 322 mothers/caretakers of children aged 12–23 months were interviewed and fortunately the nonresponse rate was zero percent. The majority, 149 (46.3%), of respondents belong to Bench ethnic group. Most, 109 (33.9%), of them were followers of Orthodox Christian religion. Majority, 220 (68.3%), of the mothers/caretakers were married. The median age of the respondents was 29.2 years, which ranges from 18 to 41 years. From the total respondents, 171 (53.1%) of mothers/caretakers did not attend any formal education. The average family size of the study population was 5.15 ranging from 2 to 11, in which most, 37%, families had less than 5 members (Table 1).

### 4.2. Sociodemographic Characteristics of the Index Child

A total of 322 children of aged 12–23 months/caretakers were included. The mean and median of children's age for those included in the study were 18 and 18.3 months, respectively. The numbers of male and female participants were 132 (41%) and 190 (59%), respectively. Majority, 173 (53.7%), of children were born at health institution, while 149 (46.3%) of them at home (Table 2).

### 4.3. Maternal Health Care Utilization

Majority, 185 (57.4%), of the mothers/caretakers had at least one antenatal care (ANC) follow-up during their pregnancy, of those mothers 119 (64.3%) had four visits and 53 (28.7%), and 13 (7.0%) of them had three and two visits, respectively. Majority, 179 (55.6%), of them had took one or more doses of tetanus toxoid (TT) vaccine and the rest 143 (44.4%) had not. From the total respondents, 158 (49.1%) of them had postnatal care (PNC) follow-up.

### 4.4. Availability and Accessibility of Vaccination Service

More than half, 182 (56.5%), of mothers/caretakers responded that they could reach the vaccination site within 30 minutes on foot. All of the respondents were reported that they had access to the health facility that provides immunization services and 132 (41.3%) of those respondents reported that they had access to hospital, 130 (40.1%) of them had access to health center, and 60 (18.6%) of them had access to health post.

### 4.5. Knowledge of Mothers/Caretakers on Vaccine and Vaccine-Preventable Disease (VPD)

Majority, 223 (69.3%), of mothers/caretaker had heard about immunization as a specific program. Major sources of information include radio, 104 (32.4%); health workers, 80 (24.9%); television, 79 (24.6%); school, 37 (11.5%); and friend, 21 (6.6%). Most, 212 (65.8%), of them knew that the objective of vaccinating children was to prevent disease, while 87 (27%) of them said they had no idea about the objectives of vaccination. Concerning age at which immunization begins, 17 (5.3%) and 200 (31.7%) of them reported that it should be started just after birth and after six weeks, respectively; 102 (31.7%) reported that immunization could be started at any time; and 2 (0.6%) reported that they did not know. Also regarding the question of how many sessions needed to get full immunization, the majority of them answered, 178 (55.3%), four sessions and 144 (44.7%) responded that less than four sessions are needed. Regarding the age at which children's immunization is completed, 276 (85.7%) of them responded that it ends at nine months (Table 3).

### 4.6. Immunization Coverage of Children Aged 12–23 Months

Only, 114 (35.4%) of mothers/caretakers showed the child vaccination card during the survey. From the total of 322 children aged 12–23 months selected and included in this study, 295 (91.6%) of them have taken one or more of the recommended vaccines and 27 (8.4%) were unvaccinated according to finding from card plus history. Of total vaccinated child, 136 (46.1%) of them finished all the recommended doses and 159 (53.9%) did not complete the entire doses (Table 4).

### 4.7. Immunization Coverage by Card Only

**Figure 1:**
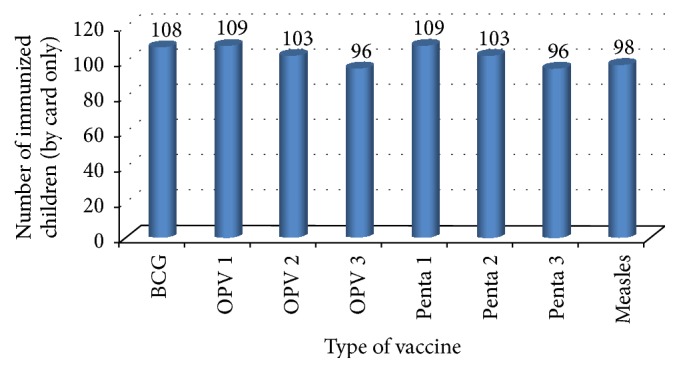
Number of immunized children aged 12–23 months for each dose of vaccine by card only at Mizan Aman town, Bench Maji zone, Southwest Ethiopia 2017.

Out of the total surveyed children aged 12–23 months, vaccination card was only seen and confirmed for 114 (35.4%) children. Coverage by card only was calculated by taking children who had vaccination card as a numerator. From 114 vaccinated by card only, 96.5% received OPV1 and penta1 followed by BCG (96.4%) and OPV2 (91.2%). Penta 3 was taken by 85% and measles vaccine was taken by 85.6% and based on the made-available vaccination card, only 81 (25.2%) children completed all the recommended vaccines (Figure 1).

### 4.8. Immunization Coverage by Card plus Mother Recall

**Figure 2:**
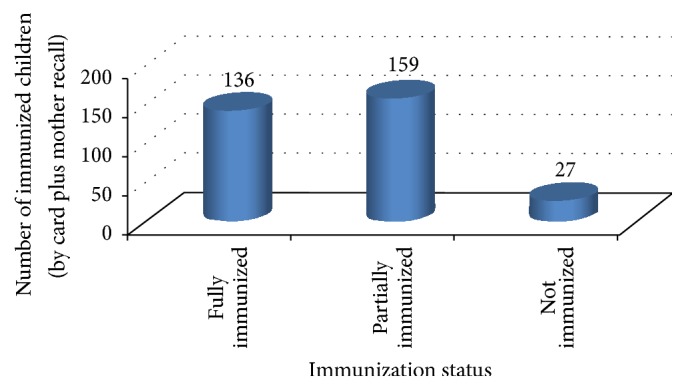
Immunization status of children aged 12–23 months by card plus mothers recall at Mizan Aman town, Bench Maji zone, Southwest Ethiopia, 2017.

Based on the vaccination card and the mother's/caretakers recall, 295 (91.6%) of the children took at least a single dose of vaccine. From the total study participants, 136 (42.2%) were claimed fully immunized, 159 (49.4%) were partially vaccinated, and 27 (8.4%) were unvaccinated (Figure 2).

### 4.9. Reasons for Vaccination Failure among Never or Partially Vaccinated Children

#### 4.9.1. Reasons for Failure to Complete Full Vaccine

The respondents who were not completing their children's vaccination were asked for reasons of failure. Accordingly, the majority, (41.8%), of them replied that forgetting the appointment date was the root cause for not completing immunization. Also 34.2% provided lack of awareness and the rest, 1.2%, of them provided absence of health worker on health facility on the day of appointment as a main reason for vaccine dropout.

#### 4.9.2. Reasons for Not Ever Vaccinating Their Child

Concerning the reason for not ever vaccinating their child, out of the total children who were not ever vaccinated, the majority (44.8%) of them replied fear of the side effects of vaccination as a cause. And 31%, 20.7%, and 3.4% of respondents replied that lack of awareness, their belief that vaccination has no benefit, and religious and custom restriction are major causes for not ever vaccinating their child, respectively.

### 4.10. Factors Associated with Child Immunization Status

In order to determine factors associated with child immunization status, both bivariate and multivariate analyses were performed. Variables, which showed a significant association, both in bivariate and in multivariate regression analysis, with immunization completion include maternal education, occupational status of the mother, place of index child delivery, ANC follow-up during pregnancy, and mothers' knowledge towards vaccine-preventable disease (Table 5).

### 4.11. Availability and Accessibility of Vaccination Service versus Completion of Immunization

The associations of health care availability and accessibility with the completion of vaccination also can be seen by using bivariate analysis. The finding from this study revealed that all children are found to have access to health facility to get vaccination service. According to the bivariate analysis result, type of health institution located nearby to children and time taken to reach health facility to get vaccination service have showed no association with full immunization of children.

## 5. Discussion

Vaccination is the centerpiece of preventive care of the well child and vaccination coverage remains an important indicator of child health outcomes in all countries. Vaccination has been one of the singular public health successes of the past half century. However until a recent time, a number of factors have been hindering the attainment of targets to provide complete vaccination in different nations for all individuals who are in need. Thus improving vaccination coverage has become the most desire of all nations to alleviate the undesirable health outcomes of nonimmunized children. In spite of observable progress in addressing immunization service globally, still now immunization coverage is not sufficient enough in contrast to its immense advantage. So this study tried to assess immunization coverage and factors associated with under vaccination among children aged 12 to 23 months in Mizan Aman town. In this study, a total of 322 mothers/caretakers were interviewed and fortunately the response rate was 100%.

Based on the finding of this study, out of total children surveyed by card plus history, the coverage of fully immunization observed was 136 (42.2%), whereas more than half (57.8%) of children did not complete the recommended immunization schedule. This figure was low compared with different study that was conducted in various regions including Istanbul [13] and Mali [14] where the proportion of fully immunized children aged 12–23 months was 84.5% and 59.9%, respectively. It was also much lower than a survey done in Illubabor zone where immunization coverage was 65.6% [10]. These differences may be attributed to forgetting the appointment date and lack of awareness by mothers/caretakers and possibly absence of health worker on health facility on the day of appointment in this study area.

Another way used to get information about immunization status of children in this method was asking mothers to show infant immunization card. According to the finding obtained from this study, out of the total interviewed mothers/caretakers, 114 (35.2%) of them showed vaccination card of their children. From these, 96.4% of children took BCG vaccines and 78.1% had taken all the recommended immunization. The immunization coverage for BCG, Penta, and OPV was above 85%. This result was better than a result obtained in Oromia regional state [15]. Also this study finding was found to be in better condition than a finding obtained in Somali land where out of total respondents only 18% of them showed vaccination card and overall coverage by card only was reported to be BCG 40.9%; OPV3 9.0% [16]. This might have happened due to variability of study participants in different settings.

According to bivariate and multivariate logistic regression analysis, maternal education, father's education, and mothers/caretakers knowledge status were found to be predictor of full immunization. This finding was congruent with a result obtained in Oromia regional state, Ethiopia [15], but it was different from the study done in Ambo Woreda, where only occupation of the mother showed significant association with the immunization status of the children [9]. This study also identified that those children whose fathers were literate had 2.42 times more likely to be fully immunized than those of illiterate. This could be associated with father's ability to make decision based on evidence because most of them had awareness on vaccine and vaccine-preventable diseases. Also it is a fact that as educational status of father increased, health seeking behavior could be increased which might lead them to vaccinate their children.

According to the finding from this study there was no evidence to support that sex of child and birth order had any impact on full immunization of children in this study. In some societies with cultural discrimination against female children, boys have a greater chance to be vaccinated. Research done in urban Bangladesh had revealed that sex and birth order had an association with child immunization status [17].

It is a fact that the place of delivery was an important determinant factor of the decision to fully immunize the child and of the coverage of immunization. This study had revealed that place of delivery of the index child showed a significant association; children who were born at health institution were [AOR = 2.4 (95% CI: 1.38, 3.65)] more likely to be fully vaccinated than those who were born at home. This finding was coherent with a result obtained from Kenya where being born in hospital increased the probability of the child being immunized by 1.5 percentage points [18]. These findings reveal that if delivery occurs in health care facility, some vaccines such as BCG are normally administered which increases the likelihood of the child being immunized which in turn amplifies the immunization coverage. Additionally mother who delivers in a hospital is more likely to receive training on benefits of immunization from health service providers.

The analysis of the regression results also reveals that receiving of the antenatal care visits was an important determinant factor of the decision to immunize children. This study revealed that children whose mothers had antenatal care (ANC) follow-up were more likely to be fully vaccinated than who did not attend ANC. This finding is consistent with that of a finding obtained in Ambo district and Mali [14] and a finding in Kenya [18]. This could have happened due to mothers health seeking behavior and mothers may discuss with health professional the vaccine and vaccine-preventable diseases, importance of immunization, time of vaccine initiation and when vaccine is completed, and possible side effect associated with vaccine. So, it may create a good opportunity for the mother to vaccinate their children.

Knowledge of mothers on immunization and vaccine-preventable disease known by the respondents was associated with full immunization of children in this area. It was identified that children whose mothers had good knowledge on immunization and vaccine-preventable disease were 6.18 more likely to be fully vaccinated than children whose mother has poor knowledge. This kind of knowledge can change mothers' health seeking behavior which in turn enhances immunization coverage of a given area. This study is consistent with study done in Oromia region Ambo district [9] and Hosanna town [19] where knowledge of mothers towards immunization contributed to vaccinating their children.

Different variables were identified as the main factor for not completing immunization in this study area. It was found out that 41.8% of them mentioned forgetting the appointment date as a cause, and others mentioned lack of awareness (34.2%) and absence of health worker on health facility on the day of appointment, 1.2%, as a factor for not completing immunization. Also concerning the reason for not ever vaccinating their child, the majority, 44.8%, of them replied that fear of the side effects of vaccination is cause and religious and custom restriction was also reported as a restricting factor. This finding was coherent with a finding obtained in Sinana district [20].

## 6. **Strength and Limitations of the Study**

### 6.1. Strength of the Study

Since there was shortage of data related to immunization coverage and factors associated with under vaccine in this area, this study might be an additional resource for those stakeholders who want to undertake any further intervention.

### 6.2. Limitations of the Study

Regarding limitations of this study, immunization coverage might have been underreported or overreported by mothers/caretakers because mothers may not remember doses that the child took due to recall bias. As a consequence, recall bias may affect quality of data. Also there might be the potential for selection bias while selecting households and choosing children from those households with more than one child during data collection time. The immunization coverage might be underreported by this study due to exclusion of non-long-term residents in this area.

## 7. Conclusion

The finding from this study revealed that child immunization coverage in the studied area was low. Occupation of mothers/caretakers, educational level of father and mother, knowledge on vaccine and vaccine-preventable disease, antenatal care follow-up, postnatal care service utilization, TT vaccination, birth order, and place of delivery of the index child were statistically significant predictors of full immunization of children.

The main reasons described for not completing immunization by respondents were forgetting the appointment date and lack of awareness of immunization. Also as a reason for not vaccinating their child, most respondents replied that vaccination hurts their child and they have low awareness about vaccination which led to undervaccination. Also some of them considered vaccination as nonessential while others mentioned religion and custom as a major factor for not immunizing their child.

## 8. Recommendation

The study's finding showed that there was a low coverage of immunization in this area. Thus the town health office and other concerned stakeholders need to be engaged in various activities to improve performance of expanded program on immunization in this area and as soon as possible to reduce the risk of vaccine-preventable diseases. The health bureau should strengthen defaulters tracing mechanism using urban health extension program workers by creating referral linkage with health facilities.

Furthermore, the health office and health facilities in town should work on reasons provided by the mothers/caregivers for vaccination incompletion such as lack of awareness of vaccination and vaccine-preventable disease, absence of trained health worker, and other factors.

Also it was better that health facility that provides EPI services should strengthen continuous staff motivation, regular supervision, and continuous monitoring and evaluation to detect decline in vaccination coverage very early. It is also important that health extension workers encourage mothers to have ANC follow-up and institutional delivery and they should discuss immunization with mothers one-to-one.

## Figures and Tables

**Table 1:** Sociodemographic characteristics of the respondents at Mizan Aman town, Bench Maji zone, Southwest Ethiopia, 2017.

Variables	Category	Frequency	Percent (%)
Marital status of the mothers/caretakers	Unmarried	1	0.3
	Divorced	76	23.6
	Married	220	68.3
	Widowed	25	7.8

Educational level of the mother/caretakers	Illiterate	171	53.1
	read and write	110	34.2
	>college	41	12.7

Occupation of the mothers/caretakers	Housewife	161	50.0
	Government employee	113	35.1
	Merchant	33	10.2
	Daily laborer	15	4.7

Ethnicity of the mothers/caretakers	Bench	149	46.3
	Kaffa	56	17.4
	Amhara	72	22.4
	Tigre	23	7.1
	Other	22	6.8

Religion of the mothers/caretakers	Orthodox	109	33.9
	Muslim	104	32.3
	Protestant	100	31.1
	Other	9	2.7

Age of the mothers/caretakers	18–24	45	14.0
	25–31	173	53.7
	32–38	101	31.4
	>39	3	0.9

Educational level of the father	Illiterate	101	31.4
	Read and write	165	51.2
	>college	56	17.4

Occupation of the father	Unemployed	80	24.9
	Farmer	154	47.8
	Government employee	48	14.9
	Merchant	20	6.2
	Daily laborer	1	0.3
	Other	19	5.9

Age of the father	18–24	39	12.1
	25–31	141	43.8
	32–38	138	42.9
	>39	4	1.2

Family size	2–4	17	5.3
	5–7	156	48.3
	>7	149	46.4

Number of children	1–3	95	29.5
	4–6	145	45.0
	>7	82	25.5

Family income	<1000	15	4.7
	1100–3000	106	32.9
	3100–5000	120	37.3
	5100–7000	72	22.3
	>7000	9	2.8

**Table 2:** Sociodemographic characteristics of children aged 12–23 months at Mizan Aman town, Bench Maji zone, Southwest Ethiopia, 2017.

Variable	Category	Frequency	Percent (%)
Sex	Male	132	41.0
	Female	190	59.0

Age (month)	12–15	107	33.2
	16–19	150	46.6
	20–23	65	20.2

Birth order	1–3	94	29.2
	4–7	146	45.3
	>7	82	25.5

Place of delivery	Home	149	46.3
	Health facility	173	53.7

**Table 3:** Respondents knowledge on vaccination and vaccine-preventable diseases at Mizan Aman town, Bench Maji zone, Southwest Ethiopia, 2017.

Variable	Category	Frequency	Percent (%)
Heard about vaccination and vaccine preventable disease	Yes	223	69.3
	No	99	30.7

Source of information	Friend	21	6.5
	Television	79	24.5
	Radio	104	32.3
	School	37	11.5
	Health worker	80	24.8
	Other	1	0.3

Objectives of vaccination	To prevent disease	212	65.8
	For healthy child	6	1.9
	It has no benefit	17	5.3
	Do not know	87	27.0

Number of vaccine-preventable diseases known	Know one	19	5.9
	Know two	56	17.4
	Know three	89	27.6
	Know four	68	21.1
	Know five	1	0.3
	I do not know	89	27.7

The age at which child start routine EPI service	Just after birth	17	5.3
	6 weeks after	200	62.1
	Any time	102	31.7
	After one year	1	0.3
	Do not know	2	0.6

Number of sessions required to complete routine EPI services	One	11	3.4
	Two	87	27.0
	Three	45	14.0
	Four	178	55.3

The age at which child completes routine EPI services	6–8 month	45	14.0
	9 month	276	85.7

**Table 4:** Immunization coverage of children at Mizan Aman town, Bench Maji zone, Southwest Ethiopia, 2017.

Variable	Category	Frequency	Percent (%)
Vaccinated	Yes	295	91.6
	No	27	8.4

Vaccination card (*n* = 295)	Yes	114	38.6
	No	181	61.4

Immunization status by card plus history	Fully vaccinated	136	42.2
	Partially vaccinated	159	49.4
	Unvaccinated	27	8.4

Immunization coverage by card only	Fully vaccinated	81	27.5
	Partially vaccinated	33	11.2
	Unvaccinated	181	61.3

Vaccination record from card	BCG	108	96.4
	OPV 1	109	96.5
	OPV 2	103	91.2
	OPV 3	96	85.0
	PENTA 1	109	96.5
	PENTA 2	103	91.2
	PENTA 3	96	85.0
	Measles	98	85.6

**Table 5:** Completion of immunization by sociodemographic characteristics of the mother at Mizan Aman town, Bench Maji zone, Southwest Ethiopia, 2017.

Variable	Category	Fully vaccinated		^*∗*^COR (95% CI)	^*∗*^AOR (95% CI)
		Yes	No		
Relation with the child	Mother	129	173	1	
	Caretaker	7	13	0.7 (0.28, 1.86)	

Maternal educational level	Illiterate	51	120	1	1
	Read and write	63	47	3.1 (1.91, 5.20)	0.6 (0.28, 1.50)
	>college	22	19	2.7 (1.33, 2.29)	1.0 (0.41, 2.45)

Maternal occupation	Housewife	46	115	1	1
	Civil servant	66	47	3.5 (2.11, 5.82)	3.14 (1.34, 3.55)
	Merchant	19	14	3.3 (1.57, 7.33)	0.4 (0.11, 1.63)
	Day laborer	5	10	1.2 (0.40, 3.85)	0.4 (0.10, 1.94)

Educational status of father	Illiterate	30	71	1	1
	Read and write	101	64	2.42 (1.27, 1.64)	0.8 (4.14, 5.55)
	>college	35	21	3.34 (1.12, 1.93)	1.0 (0.44, 2.33)

Place of index child birth	Health institution	84	89	2.2 (1.46, 3.63)	2.4 (1.38, 3.67)
	Home	47	102	1	1

Antenatal service (ANC) service utilization	Yes	105	80	1	1
	No	31	106	0.2 (0.13, 0.36)	0.3 (0.05, 2.83)

Tetanus toxoid (TT) vaccination	No	34	109	1	1
	Yes	102	77	4.2 (2.62, 6.92)	1.2 (0.18, 9.11)

Postnatal care (PNC) visit	Yes	82	76	2.2 (1.40, 3.45)	1.6 (0.65, 4.13)
	No	54	110	1	1

Knowledge on vaccine and vaccine preventable disease (VPD)	Good knowledge	121	89	8.7 (4.73, 16.11)	6.18 (3.0, 12.6)
	Poor knowledge	15	97	1	1

^*∗*^ *COR, crude odds ratio; AOR, adjusted odds ratio*.
